# An electrochemical, and surface studies of synthesized Gemini ionic liquid as corrosion inhibitor for carbon steel in petroleum field

**DOI:** 10.1038/s41598-024-58321-2

**Published:** 2024-05-10

**Authors:** Yousef A. Selim, M. Abd-El-Raouf, K. Zakaria, Ahmed Z. Sayed, Yasser M. Moustafa, Ashraf M. Ashmawy

**Affiliations:** 1 Chemistry Department, Faculty of Science, Al-Azhar University, Nasr City, 11884, Cairo, Egypt; 2 Egyptian Petroleum Research Institute (EPRI), Nasr City, P.O. 11727, Cairo, Egypt

**Keywords:** Ionic liquids, Electrochemistry, Corrosion inhibitor, Carbon steel, Electrochemistry, Corrosion

## Abstract

In this work, we study the efficiency of *N*^1^, *N*^3^-dibenzyl-*N*^1^, N^1^, *N*^3^, *N*^3^-tetramethylpropane-1,3-diaminium chloride, as anticorrosion. This compound exhibits potential as a prospective remedy to stop the deterioration of carbon steel caused by corrosion in 1.0 M HCl. The synthesis of this compound is described in a comprehensive manner, and its composition is supported by a range of precise analytical approaches such as elemental analysis, and mass spectroscopy. Based on the findings of the investigation, the synthesized Gemini ionic liquid demonstrates a robust capacity to slow down the rate at which the metal corrodes. The Prepared compound was evaluation by electrochemical and morphology study. Our results revealed that elevating the inhibitor concentration led to an augmentation in inhibition effectiveness, reaching up to 94.8% at 200 ppm of the synthesized compound at 298 K. It is crucial to emphasize that the recently prepared Gemini ionic liquid is consistent with the Langmuir adsorption model and function as a mixed inhibitor, participating in the physio-chemisorption process of adsorption.

## Introduction

Corrosion is widely recognized as a significant concern in a range of engineering fields, including metallurgy, mechatronics, chemical engineering, automotive engineering, medical engineering, and construction. Its detrimental effects can have severe implications for safety, preservation, and economic considerations^1,2^. The petroleum industry is a rapidly expanding sector within the industrial domain that is very susceptible to corrosion-related challenges. Corrosion significantly impacts all facets of exploration and production, encompassing offshore rigs and casing, resulting in substantial financial losses up to billions of dollars for nations on a yearly basis. Metal properties, mechanical variables, electrical potential difference, temperature, pH, and circumstances outside are all contributors to corrosion. Acids, bases, salts, moisture, pollutants, and other corrosive elements are all part of the environment were talking about here. The interplay of these factors influences both the extent and speed of metal corrosion^3–5^.

Ongoing research is being conducted to identify optimal strategies for mitigating the impact of corrosion. Various techniques have been utilized to minimize the effects of corrosion, including the application of metallic and organic protective coatings, The utilization of metals that possess resistance to corrosion, the implementation of cathodic protection, the utilization of polymers and plastics, and the incorporation of corrosion inhibitors^6^. The utilization of inhibitors of corrosion is often regarded as a very effective and economically advantageous strategy for mitigating corrosion, particularly within the oil and gas sector^7^. Organic compounds possess multiple adsorption active sites, including atoms such as N, O and S. These compounds are crucial inhibitors that effectively mitigate industrial corrosion issues and significantly contribute to the mitigation of their adverse effects^8–12^. Despite their effectiveness, the majority of organic chemicals exhibit several significant limitations, such as high cost and toxicity towards living organisms^13^.

Nevertheless, a significant portion of the existing scholarly literature focuses on the investigation of corrosion and scale inhibitors that are derived from medicinal medicines, extracts of plants, and ionic solutions. These chemical green inhibitors contain excellent features, including environmental friendliness, easy accessibility, biodegradability, non-toxicity, and affordability^14–18^. In recent studies, it has been established that ionic liquids (ILs) possess notable effectiveness and exhibit potential as environmentally friendly inhibitors for corrosion in diverse industrial metals and alloys, particularly in corrosive environments characterized by saline, alkaline, or acidic solutions. This can be attributed to the substantial size of ILs' molecular structure and the inclusion of hetero-atoms^19–22^. Consequently, the focus of this study revolves around the advancement of a newly formulated ionic liquid compound; the chemical compound has been formulated and synthesized with the purpose of serving as a corrosion inhibitor specifically designed for application in conjunction with carbon steel materials. The substance's major function is to reduce corrosion in a very acidic setting analogous to that seen in petroleum fields.

## Experimental

### Synthesis of Gemini ionic liquid compound

**Figure 1:**
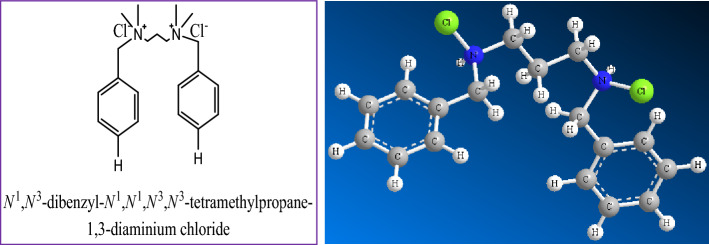
The chemical and optimized molecular structure of the synthesized Gemini ionic liquid compound.

**Figure 2:**
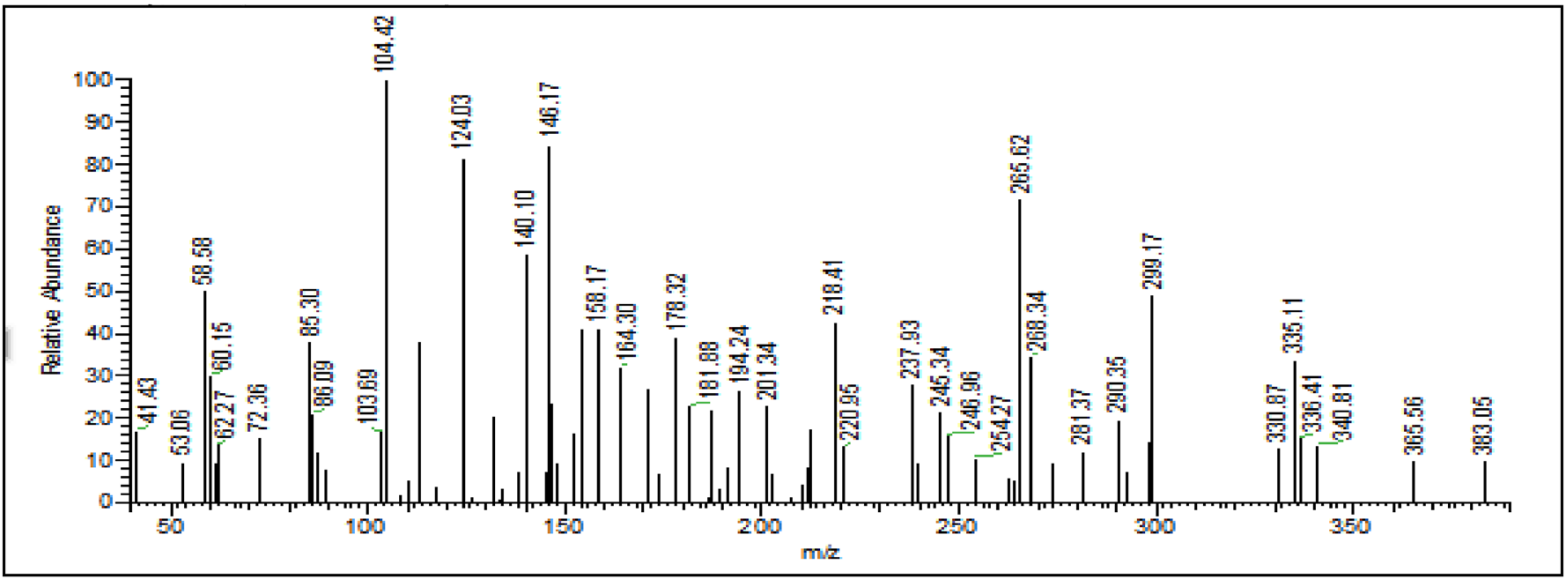
Mass spectroscopy chart of the prepared Gemini ionic liquid.

The Gemini ionic liquid inhibitor was prepared according to the previous described work elsewhere^23^. The chemical structure of the synthesized Gemini ionic liquid is shown in Fig. 1. The chemical structure was brought to light by several technical methods. Moreover, a mass spectrum was composed using a Thermo Scientific Gcms Model—Japan at the Regional Center for Mycology and Biotechnology (RCMB), Al-Azhar University in Nasr City, Cairo, Egypt to estimate the compounds average fragmentation and molecular weight, as well as evaluate the compounds' purity through the instrument's direct intake. The mass fragmentation of the GIL-H is illustrated in Fig. 2. The determined mean molecular weights of the Gemini ionic liquid derivative product are given in Table 1 have been found to be very near to that calculated theoretically.

**Table 1:** The theoretically and determined molecular weights of the prepared ionic liquid compound.

Atom, %	Theoretical	Determined
C	65.79	65.61
H	8.41	8.58
N	7.31	7.59

### Aggressive medium

Analytical grade HCl with a concentration of 37% (E. Merck) was appropriately diluted using distilled water. Put together a remedy with a corrosive nature and a specific concentration, 1.0 M HCl. The Gemini ionic liquid compound was produced and subsequently employed at concentrations of 25, 50, 100, and 200 ppm.

### Metal specimen composition

Electrochemical studies were carried out on a carbon steel metal (G 60) having a cylindrical shape embedded in epoxy resin with an exposed surface area of 7.5 mm diameter to the electrolyte serving as working electrode. The insinuation area was abraded with a series of emery paper (180-320-600-800-1000-2000-2500) and then washed with bi-distilled water and acetone before running out all experiments. The elemental chemical composition of the investigated carbon steel is shown in Table 2.

**Table 2:** The chemical composition of carbon steel.

C	Si	Mn	P	S	Ni	Cr	Al	V	Ti	Cu	Fe
0.19	0.005	0.94	0.009	0.004	0.014	0.009	0.034	0.016	0.003	0.022	Rest

### Electrochemical measurements

The electrochemical experiment utilized a glass cell with three terminals. The operational electrode utilized in the experiment was composed of carbon steel, whilst the reference electrode consisted of a platinum wire. Additionally, the auxiliary electrode employed was a saturated calomel electrode (SCE). All the readings were taken at room temperature using a potentiostat and galvanostat from the French company Origalys; model number Origa Flex-Pack OGF01A. Origamaster 5 version 2-4-0-4 was used as the system controller. A steady-state open circuit potential (E_ocp_) used to programmatically alter the test's potential to fail from − 700 to − 300 mV versus the standard calomel electrode (SCE), keeping the scan rate constant at 1 mV/sec. This adjustment was made to facilitate the generation of potentiodynamic polarization (PDP) curves. The initiation of electrochemical testing was delayed until the working electrode had been submerged in the test fluids for duration of 15 min. It was during this period that the steady-state open circuit potential (Eocp) could be ascertained.

Electrochemical impedance spectroscopy (EIS) measurements were taken across a frequency range of 10^5^ to 10^–2^ Hz as part of the experimental process. We did this by adding 10 at regular intervals based on frequency. The measurements were taken at the open circuit potential (E_ocp_) under steady-state circumstances after the devices had been submerged in test solutions for 15 min. Both solutions, with and without Gemini ionic liquid (GIL) inhibitor, were used to conduct the experiments. A 10 mV peak-to-peak alternating current (AC) was used as the excitation signal in this study. 298 K was used for the electrochemical testing and bode, and Nyquist plots were used to display the electrochemical impedance spectroscopy (EIS) results^24^.

### Surface morphological examinations

#### Scanning electron microscopy SEM/energy-dispersive X-ray spectroscopy EDX and X-ray photoelectric spectroscopy XPS

When moving from untreated solutions to solutions comprising varied doses of inhibitors, the method of assessing the surfaces of carbon steel changed significantly. The use of “energy-dispersive X-ray spectroscopy (EDX)” was employed in the present work for the characterization of the surface constituents of carbon steel. Scanning electron microscopy (SEM) with a JEOL/JSM/6510 model, connected to an EDX Unit with a 30 kV accelerating voltage, a magnification range of 14 to 1,000,000, was used in an accompanying investigation to examine surface morphological alterations. A full treatment was performed on the carbon steel samples by immersing them in a 1.0 M HCl solution for 24 h prior to the tests being performed. The preparation technique was carried out at a constant 298 K before and after the addition of various amounts of inhibitor. X-ray photoelectron spectroscopy (XPS) investigation was conducted utilizing equipment from Thermo Fisher Scientific, USA. Al Kα radiation with an energy of 1350 eV was employed for the measurements. The emission voltage and power of the X-ray source were configured at 11 kV and 220 W, respectively. The analyzing chamber maintained a constant pressure of 10^–9^ mbar during the entire analysis^25^.

#### AFM

The research employed the Flexaxiom Nanosurf C3000 Atomic Force Microscopy (AFM) model to analyze the roughness of the surface and three-dimensional morphology of carbon steel. The experimental procedure employed in this study involved the utilization of Dynamic Mode, utilizing a Cantilever Resonance Frequency of 9 Hz. The investigation was conducted following the immersion of the carbon steel in a 1.0 M HCl solution for duration of 24 h. The research encompassed two experimental conditions: one in which no inhibitor was present, and another in which inhibitor was introduced at 200 ppm concentration of the GIL-H.

### Computational procedure

As the corrosion process investigated herein in acidic media, the examined inhibitor was optimized, and its quantum chemical indices were estimated using DFT utilizing BIOVIA Materials Studio 6.0 (17.1.0.48). DMOL_3_ module has been created using the Generalized Gradient Approximation (GGA) and group functional basis designed to take into consideration its chemical reactivity using medium quality tolerance parameters, a Becke One Parameter (BOP) and Double Numerical plus Polarization (DNP-3.5). Energies (E_HOMO_ and E_LUMO_) of the Highest Occupied Molecular Orbital (HOMO) and that of the Lowest Unoccupied Molecular Orbital (LUMO), energy gap in eV (ΔE = E_LUMO_—E_HOMO_), electronegativity (χ) and fraction of the electron transferred (∆N) were computed^26^.

## Results and discussion

### Open circuit potential

**Figure 3:**
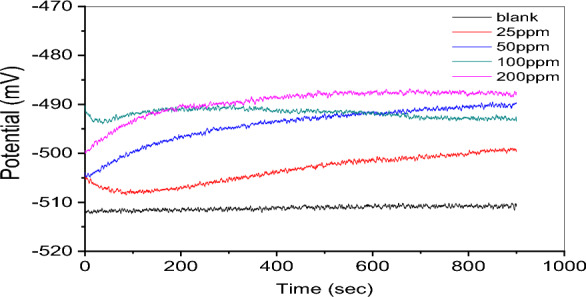
Potential-time curves (OCP) for carbon steel in 1.0 M HCl at different concentrations of the new Gemini ionic liquid inhibitor.

Figure 3 depicts the open-circuit potential (OCP) changes of carbon steel after being immersed in a 1.0 M HCl solution at 298 k while in free corrosion circumstances. The experiment was run with and without the synthesized Gemini ionic liquid (GIL-H). After a certain amount of time had passed in seconds, the OCP values were recorded. The open circuit potential (OCP) measurements conducted in an inhibitor-free solution exhibit a discernible pattern characterized by increasingly negative results. The electrode potential remains steady at approximately − 512 mV compared to the Ag/AgCl reference electrode throughout the whole immersion period. The findings presented in this study provide support for the assertions put out in citation, which propose that the carbon steel's surface has seen the deposition of corrosion products^27^.

Nevertheless, when varying quantities of inhibitor molecules are introduced, the measured potential undergoes an initial positive change. When an inhibitor is present, the initial open circuit potential (OCP) is much higher than it would be in a blank solution. At high concentrations of GIL-H (200 ppm), this is accompanied by a large negative shift to around − 486 mV after 900 s. For all inhibitor concentrations, a transition to a stable state occurs after about 7 min, and this is explained by a change in the interface surface. It is believed that inhibitor molecules adsorb at corrosion sites on the surface of carbon steel, creating a thermodynamically more stable state^28^.

### Potentiodynamic polarization (PDP) curves

**Figure 4:**
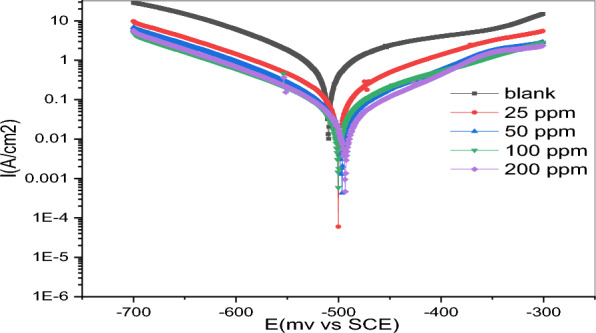
Potentiodynamic polarization curves for carbon steel in 1.0 M HCl in the absence and presence of various concentrations of GIL at 298 K.

The objective of this investigation was to assess the influence of a synthetic Gemini ionic liquid on the corrosion characteristics of carbon steel when exposed to a solution of hydrochloric acid with a concentration of 1.0 M. Figure 4 shows the polarization graphs for the Gemini ionic liquid that was analyzed at a temperature of 298 k. The impact of the Gemini Ionic liquid inhibitor on the anodic and cathodic reactions is shown graphically. The present discovery indicates that the incorporation of Gemini Ionic liquid inhibitor results in a reduction in the corrosion of carbon steel during the anodic phase and a delay in the initiation of the cathodic hydrogen evolution reaction. This observation suggests that the inhibitor exerts a suppressive influence on both the anodic and cathodic properties^29^.

Table 3 presents the electrochemical polarization characteristics associated with corrosion, including the (I_corr_) corrosion current density, the (E_corr_) corrosion potential, the (β_c_) cathodic Tafel slope, the (β_a_) anodic Tafel slope, and the (η_%_) inhibition efficiency. These features are typical of the corroding procedure. Using the provided Eq. (1), which factors in the density of the corrosion current, the inhibitory efficiency was calculated as a percentage^30^.

1 $$ \eta_{Tafel} ,\,\% = \left( {1 - \frac{i}{{i_{o} }}} \right) \times 100. $$

**Table 3:** Polarization parameters and the corresponding inhibition Efficiencies for the corrosion of carbon steel in 1.0 M HCl containing different concentrations of GIL-H at 298 K.

Conc., ppm	E_corr._, (mV)	I_corr._, (mA/cm^2^) ± SD	β_a_, (mV/dec) ± SD	β_c_, (mV/dec) ± SD	R_p_, (Ώ cm^2^)	C_R_ (mm/y)	Ɵ	η_Tafel,_ (%)
Blank	− 509.8	1.6042 ± 3.1	278.8 ± 2.8	− 144.5 ± 3.1	24.31	16.51	–	–
25	− 500.1	0.2828 ± 2.4	141.5 ± 3.2	− 132.6 ± 1.8	129.19	8.17	0.824	82.4
50	− 496.9	0.1332 ± 6.1	131.8 ± 4.1	− 119.7 ± 2.7	272.46	3.30	0.917	91.7
100	− 500.2	0.0888 ± 3.4	131.1 ± 3.2	− 118.2 ± 3.1	287.33	1.55	0.945	94.5
200	− 493.5	0.0841 ± 5.2	112.4 ± 2.8	− 116.2 ± 2.1	385.26	0.983	0.948	94.8

In the absence of the tested Gemini ionic liquid, the density of the corrosion current is marked by (i_°_), while in its presence, it is represented by (i). The polarization resistance (R_p_) was determined using the conventional Stern-Geary equation. The Tafel extrapolation approach was employed to perform this computation, where the Tafel slopes for the anodic and cathodic reactions, Eq. (2), were denoted as (ß_a_) and (ß_c_) correspondingly, as reported in reference^31^.

2 $$ R_{p} = \frac{{\beta_{a} \beta_{c} }}{{2.303\left( {\beta_{a} + \beta_{c} } \right)}} \times \frac{1}{{i_{corr} }}. $$

The findings imply that the Gemini ionic liquid's ability to prevent corrosion improves with the concentration of the inhibitor within it. The concentration of 200 ppm is optimal for inhibition, reaching a maximum of 94.8% effectiveness. According to the pattern, the corrosion of carbon steel in a 1.0 M HCl can be significantly reduced by using Gemini ionic liquid as an inhibitor. Variations in the concentration of inhibitor had an impact on both the cathodic Tafel slope (β_c_) and the anodic Tafel slope (β_a_) of the Gemini ionic liquid (GIL). It is probable that both processes were regulated by the presence of inhibitor molecules. The immobility of the particles on the metal's surface impeded the functionality of the active sites, resulting in a deceleration of the corrosion process^32–34^.

Furthermore, our data indicates that the corrosion potential (E_corr_) undergoes a modest change towards more negative and positive values in the presence of an inhibitor. This discovery demonstrates that inhibitor to the metal surface through a method that obstructs both the anodic and cathodic processes. The study also observed that the (E_corr_) readings exhibited fluctuations of less than 85 mV. The ionic liquid functions as an inhibitor of mixed-type primarily affecting the cathodic process, as evidenced by the simultaneous decrease in the densities of the cathodic and anodic currents^34,35^. Inhibitor-metal-surface contact, a mechanism whose efficacy increases with increasing inhibitor concentrations, provides insight into this phenomenon. Furthermore, activation is seen to control the inhibition mechanism in the presence of the examined inhibitor. The synchronous shifts in the cathodic and anodic Tafel curves show, however, that the effect of this Gemini ionic liquid (GIL) on the metal dissolving process is unaffected.

### Electrochemical impedance spectroscopy (EIS)

EIS stands out as a precise, swift, and robust tool for probing corrosion and inhibitory mechanisms. In this study, EIS, both with and without the presence of inhibitor, was employed to scrutinize corrosion inhibition across various concentrations of the prepared GIL-H. The electrical resistance of the steel electrode immersed in 1.0 M HCl solution was measured with different concentrations (25, 50, 100, and 200 ppm) of the GIL-H inhibitor. The acquired EIS data are tabulated and deliberated upon, with Table 4 presenting the results for the characteristic parameters of the impedance diagram. The parameter R_ct_, serving as the change transfer resistance, is found to be inversely proportional to the corrosion rate. The semicircle fitting method was applied to compute the electrochemical impedance parameters, where a perfect semicircle in Nyquist plots represents a single time constant. Impedance tests provide insights into surface resistance, capacitance, the inhibitory potential of a substance, and the nature of the inhibition process. Any deviations from a perfect circular shape are attributed to interfacial impedance frequency dispersion, associated with surface non-homogeneity and metal roughness^36–39^.

**Table 4:** Electrochemical impedance parameters for the corrosion of carbon steel in the absence and presence of different concentrations of GIL-H at 298 K.

Conc., ppm	(${R}_{s}$), (Ω.cm^2^)	(${R}_{ct}$), (Ω.cm^2^) ± SD	CPE_dl_			Ɵ	η_EIS_ (%)
			(${C}_{dl}$), (µF/cm^2^)	Yo	n		
Blank	1.84	19.66 ± 0.0034	952.10	50.97	0.999	–	–
25	1.37	52.77 ± 0.0081	788.55	15.03	0.987	0.6274	62.74
50	1.61	63.17 ± 0.0053	819.63	13.09	0.993	0.6888	68.88
100	1.11	77.12 ± 0.0043	772.41	9.88	0.974	0.7451	74.51
200	1.53	108.48 ± 0.0031	674.28	6.09	0.971	0.8188	81.88

Our investigation yielded a pattern that was not a perfect semicircle. This could be related to the addition of an inhibitor, which completely distracts the semicircle.

**Figure 5:**
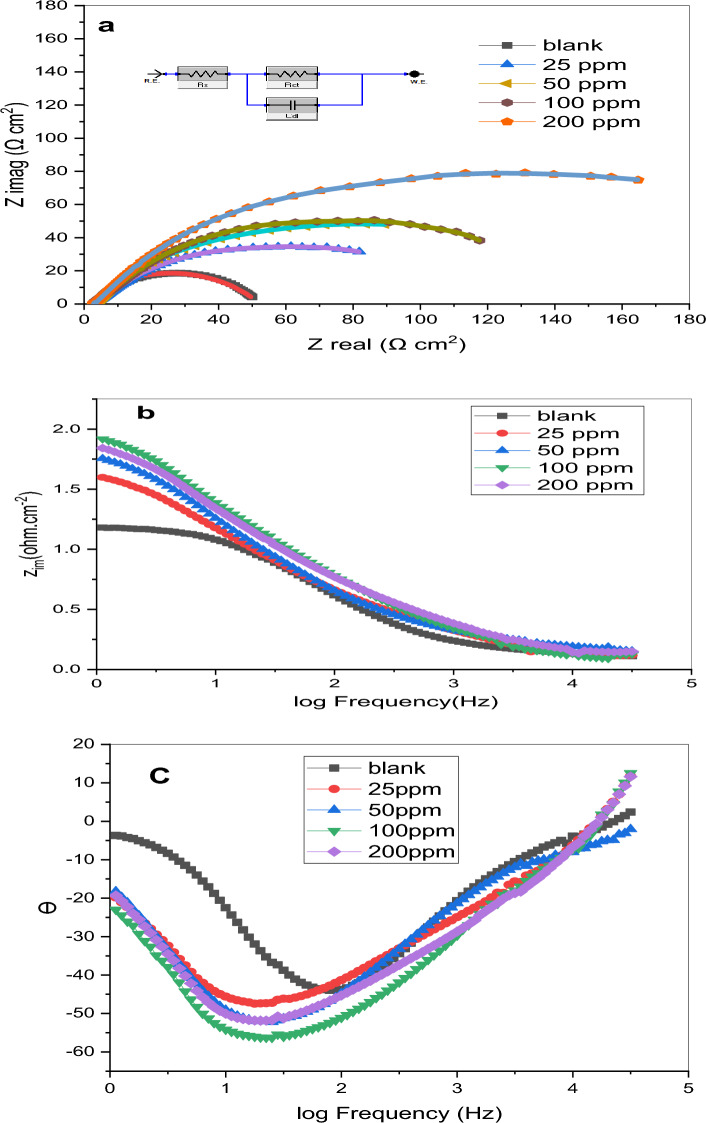
(**a**) Nyquist plots, (**b**,**c**) body plots for carbon steel in 1.0 M HCl in the absence and presence of different concentrations of GIL at 298 K.

Single semicircles are shifted in lockstep with the true impedance of the x-axis in the Nyquist plot Fig. 5a. The impedances increased as the concentrations of inhibitor increased. In addition, the impedance profiles were consistent across all concentrations. Inhibitors can alter the process by slowing down the rate of corrosion. Furthermore, the rate-controlling mechanism can be determined to be a reaction-controlled mechanism due to inhibitor corrosion^11,12^.

As inhibitor concentrations rise, R_ct_ values rise. As a result, the charge transfer mechanism oversees most corrosion responses. Furthermore, an increase in inhibitor concentration results in a decrease in C_dl_ values due to the increased surface coverage of the inhibitor. The double-layer capacitance (C_dl_) is connected in parallel to the charge transfer resistance (R_ct_) in the analogous circuit. The constant phase element (CPE) data parameters (Y_o_ and n), which represent the magnitude and an amenable parameter, respectively, can be used to analyses Cdl data^22 ,36–40^. The following equation was used to determine (C_dl_) using the CPE parameters for the circuit.

3 $$ {\text{C}}_{{{\text{dl}}}} = \, \left( {{\text{Y}}_{{\text{o}}} {\text{R}}_{{{\text{ct}}}}^{{({1} - {\text{n}})}} } \right)^{{{1}/{\text{n}}}} . $$

This is because inhibitory efficiency has increased. The decrease in the C_dl_ value could be explained by a lower local dielectric constant and/or a thicker electric double layer on the CS surface.

Due to the free mobilities of the ions, non-inhibited solution had the lowest R_ct_. According to the Rct and C_dl_ values (Table 5), GIL inhibits corrosion of CS in 1.0 M HCl via adsorption of its molecules on the surface. The following formula can be used to calculate corrosion inhibition efficiency:

4 $$ \eta_{{{\text{EIS}}}} \left( \% \right) = {\text{R}}_{{{\text{ct}}}} - {\text{R}}_{ct}^{\prime } {\text{/R}}_{{{\text{ct}}}} *{1}00, $$

where R_ct_ -R^′^_ct_ represent the charge transfer resistance in the presence and absence of GIL, respectively. The corrosion layer that develops between the grid and the active material causes a layer with a complicated structure to grow on the grid, which lowers the grid’s conductivity and raises resistance across the corrosion layer between the grid and the active material. Corrosion products have a higher specific volume due to their reduced density. Because some of the corrosion layers flake off and break the contact between the grid and the active material, there is mechanical stress and consequently a loss of active material^43^. Therefore, these findings demonstrated the ionic liquid’s significance as a corrosion inhibitor for CS.

**Table 5:** The results of various adsorption isotherm for C-steel in 1.0 M HCl with the GIL-H inhibitor determined from PDP.

	Adsorption isotherm	Equation	PDP		
			R^2^	Slope	Intercept
Inhibitor GIL-H	Flory—Huggins isotherm	log(θ/C) = log K + n log (1 − θ)	0.798	1.63	− 0.159
	Freundlich isotherm	log θ = log k + 1/n log C	0.879	− 0.067	− 0.016
	Frumkin isotherm	log θ/(1 − θ) C = log k + 2a θ	0.997	− 1.613	3.553
	Langmuir isotherm	$\frac{C}{\theta }=\frac{1}{K}+ {C}$	1.000	1.035	0.0087
	Temkin isotherm	$\uptheta =-\frac{1}{2a}{\text{ln}}C$ $-\frac{1}{2a}{\text{ln}}K$	0.986	− 1.026	5.112

## Adsorption isotherm

To cultivate a more comprehensive comprehension of the interplay between metal surfaces and ionic liquid compounds, a series of meticulous investigations were executed to delve into adsorption isotherms. To slow down the corrosion process, molecules designed to inhibit corrosion are attached to reactive spots on the outermost layer of carbon steel using the proposed analytical approach^44^. There are two basic adsorption mechanisms that can occur on the surface of carbon steel.

The initial type is referred to as physical adsorption, occurring when charged substances and surfaces with opposite charges make contact, resulting in an electrostatic interaction. The second category, recognized as chemical adsorption, occurs when a coordination bond is formed by the transfer of charges between the molecules of an ionic liquid and the surface.

Various adsorption isotherms, such as the Freundlich, Langmuir, and Temkin models, can be efficiently utilized to explicate the correlation between adsorbed species and their respective surface coverage Table 5. The examination of this correlation is performed by the application of potentiodynamic polarization (PDP) technique, which are executed under controlled conditions. The results suggest a relationship between the behavior of the inhibitor, which is made up of the Gemini ionic liquid, and the predicted results outlined in Eq. (5) of the Langmuir adsorption isotherm model^45^.

5 $$ \frac{{C_{inh} }}{\theta } = \frac{1}{{K_{ads} }} + C_{inh} $$

**Figure 6:**
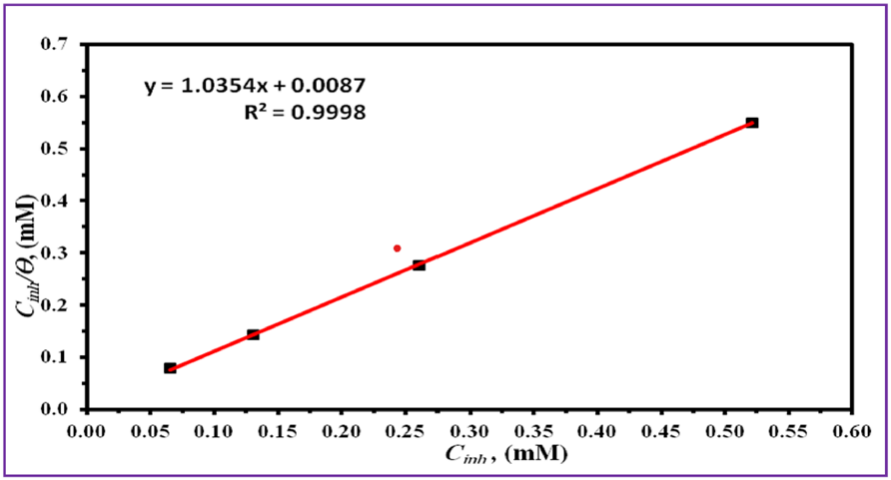
Adsorption isotherm model for investigated GIL compound at 298 K obtained from PDP.

The equilibrium constant, denoted as K_ads_, and the inhibitor concentration, denoted as C_inh_, are both found by calculating the reciprocal of the intercept. Figure 6 illustrates the linear association between the ratio of C_inh_ /θ and C_inh_. The slope of the linear function is equal to 1. The R-squared score, which exceeds 1, indicates a correlation that is very close to perfect. The slight deviation from unity is frequently ascribed to the interplay between the molecules of the Gemini ionic liquid that have formed on the surface of the metal^45^. Consequently, the Langmuir slope specific to the ionic liquid is employed for the determination of the adsorption equilibrium constant, denoted as K_ads_. The results of this investigation offer elucidation on the adherence of the Gemini ionic liquid to the Langmuir model when employed on the surface of carbon steel. The Gibbs free energy of adsorption can be calculated using the adsorption equilibrium constant (K_ads_) following the equation given below (ΔG^o^_ads_), Eq. (6).

6 $$ K_{ads} = \frac{1}{55.5}\exp \left( { - \frac{{\Delta G_{ads}^{o} }}{RT}} \right) $$

(T = kelvin temperature, R = gas constant).

The quantitative values for K_ads_ and ΔG^o^_ads_ have been meticulously computed. The range of ΔG^°^_ads_ values below − 20 kJ/mol is indicative of a predominant involvement of intermolecular forces and physical interactions driving the adsorption phenomenon. Conversely, the realm encompassing ΔG^°^_ads_ values approximating or surpassing − 40 kJ/mol is suggestive of a pronounced shift towards adsorption processes governed by chemical considerations^46,47^. Within this context, the precise ΔG^o^_ads_ value calculated in this study stands at − 31.225. This outcome substantiates, that the adsorption isotherm associated with the synthesized ionic liquid conforms to the attributes typifying physio-chemisorption phenomena.

## Surface analysis

### SEM/EDX and XPS

**Figure 7:**
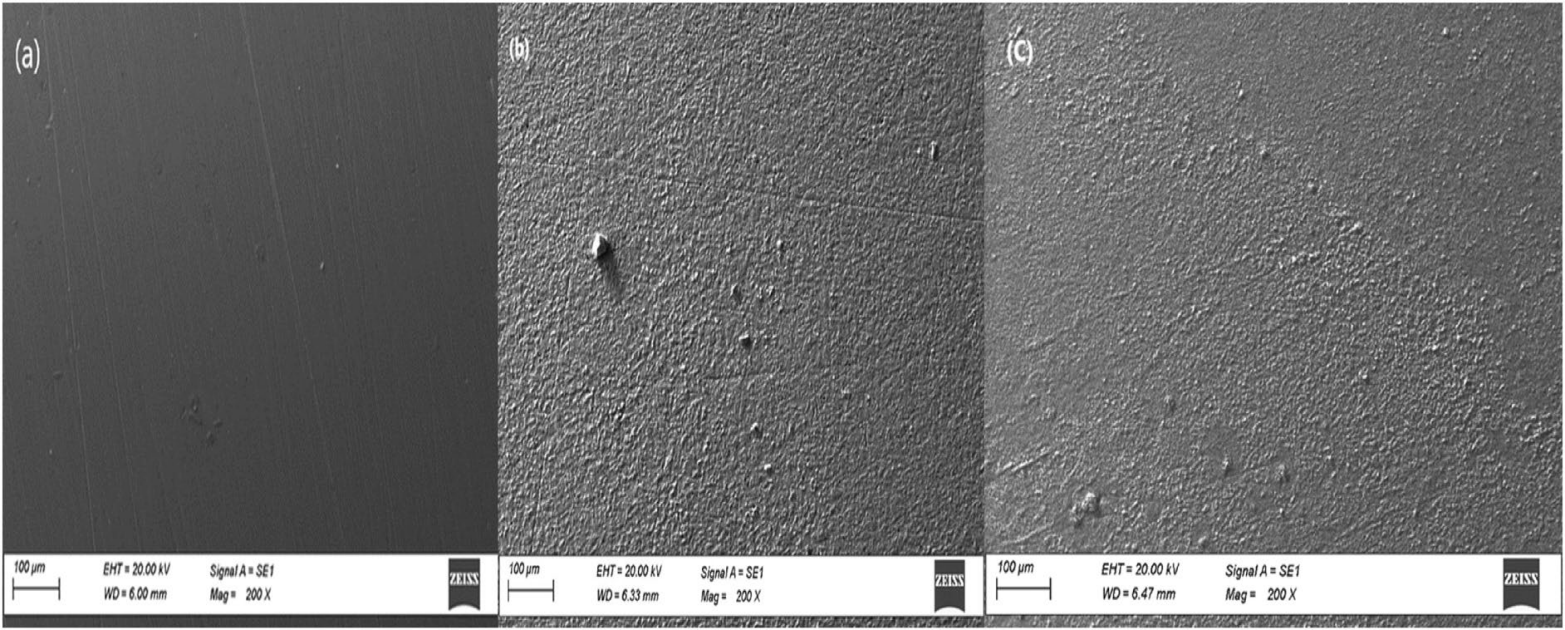
SEM micrograph pattern of carbon steel in the absence and presence of the optimum concentration of the tested GIL, (**a**) Polish (**b**) blank and (**c**) blank with 200 ppm GIL compound.

SEM analysis was performed on carbon steel samples that had been subjected to a 1.0 M HCl for 24 h. The purpose was to examine their surface properties, and both samples with inhibitor and samples without inhibitor were included in the study^48,49^. Figure 7a–c depicts the state of carbon steel after being submerged for 24 h at a temperature of 298 K in a hydrochloric acid solution with a concentration of 1.0 M for both scenarios, with and without the presence of an inhibitor. Figure 7b depicts the scanning electron microscope (SEM) image, which illustrates the surface of the carbon steel after being exposed to a 1.0 M HCl solution. The evidence demonstrates that the entire exterior of the carbon steel is typically covered by corrosion products because of significant metal loss when placed in a corrosive solution without an inhibitor. Furthermore, the porous layer of corrosion products formed is ineffective in safeguarding the surface from severe corrosion. What's captivating about Fig. 7c is that when the ideal concentration of the formulated ionic liquid inhibitor 200 ppm is introduced, the surface becomes smoother. Upon closer examination, the presence of a reduced quantity of corrosion products on the surface is clearly apparent in comparison to the untreated sample. The observation of color variation on the inhibited surface implies the development of a protective layer composed of the ionic liquid. This layer acts as a very efficient barrier on the surface^50^.

**Figure 8:**
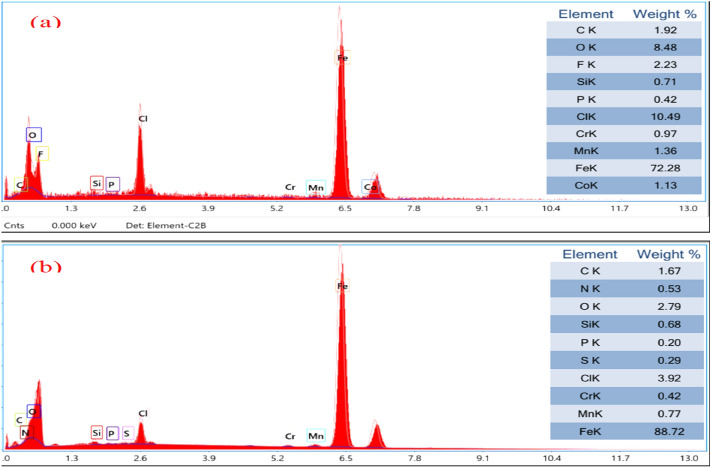
EDX spectra of carbon steel in the absence and presence of the optimum concentration of the tested GIL, (**a**) in blank and (**b**) blank with 200 ppm GIL compound.

Shifting our focus to the practical observations regarding alterations in surface morphology, we conducted EDX analysis to distinguish the different components constituting the layer generated on carbon steel when it is subjected to the examined environment. This analysis was done both when the ionic liquid compound was absent and when it was present. The EDX spectra of untreated and treated carbon steel specimens are depicted in Fig. 8. The EDX results presented in Fig. 8a illustrate the observations made on the carbon steel sample while exposed to the uninhibited solution. The analysis reveals the presence of peaks corresponding to O, Cl, and Fe. This suggests the formation of compounds such as iron oxide (FeO.nH_2_O) and/or iron chloride (FeCl_2._nH_2_O) on the surface of the metal. These findings suggest that an oxide film has formed on the carbon steel surface when inhibitors are not present. The proportion of iron (Fe, wt. %), which is 72.28, is lower than that in the inhibited system^51^. This discrepancy signifies that the carbon steel surface has undergone changes in the test environment. However, in contrast, the EDX graphs in Fig. 8b, representing the optimal configuration carbon steel with 200 ppm of IL-H, exhibit the emergence of the N peak from the ionic liquid inhibitor molecules. This implies that a layer of protective inhibitor has been applied to the surface of the carbon steel, which possesses the ability to effectively protect against the process of metal corrosion^52^. In addition, there was an observed decline in the peaks associated with O and Cl, coupled with increased levels of Fe content. This combination of factors serves to substantiate the inhibitory effects of the system. Consequently, the prepared inhibitor demonstrates the proficiency in protecting carbon steel against corrosion^53^.

**Figure 9:**
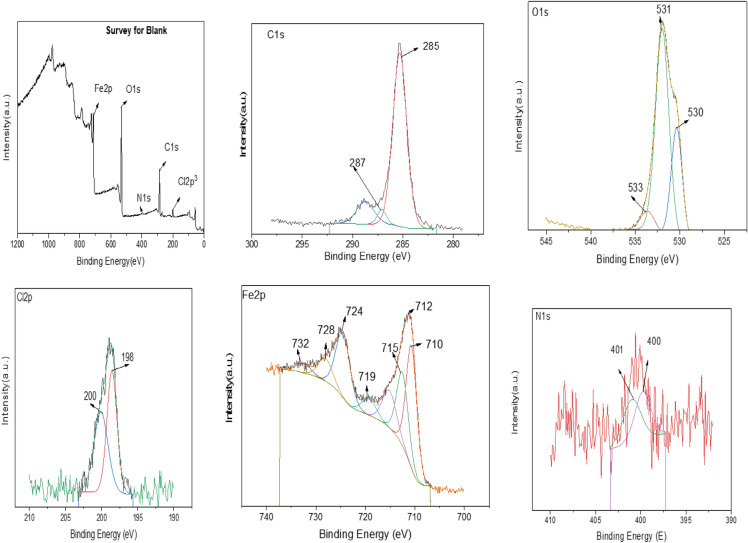
XPS spectra of the CS alloy after 24 h of immersion in 1.0 M HCl in the absence GIL inhibitor.

**Figure 10:**
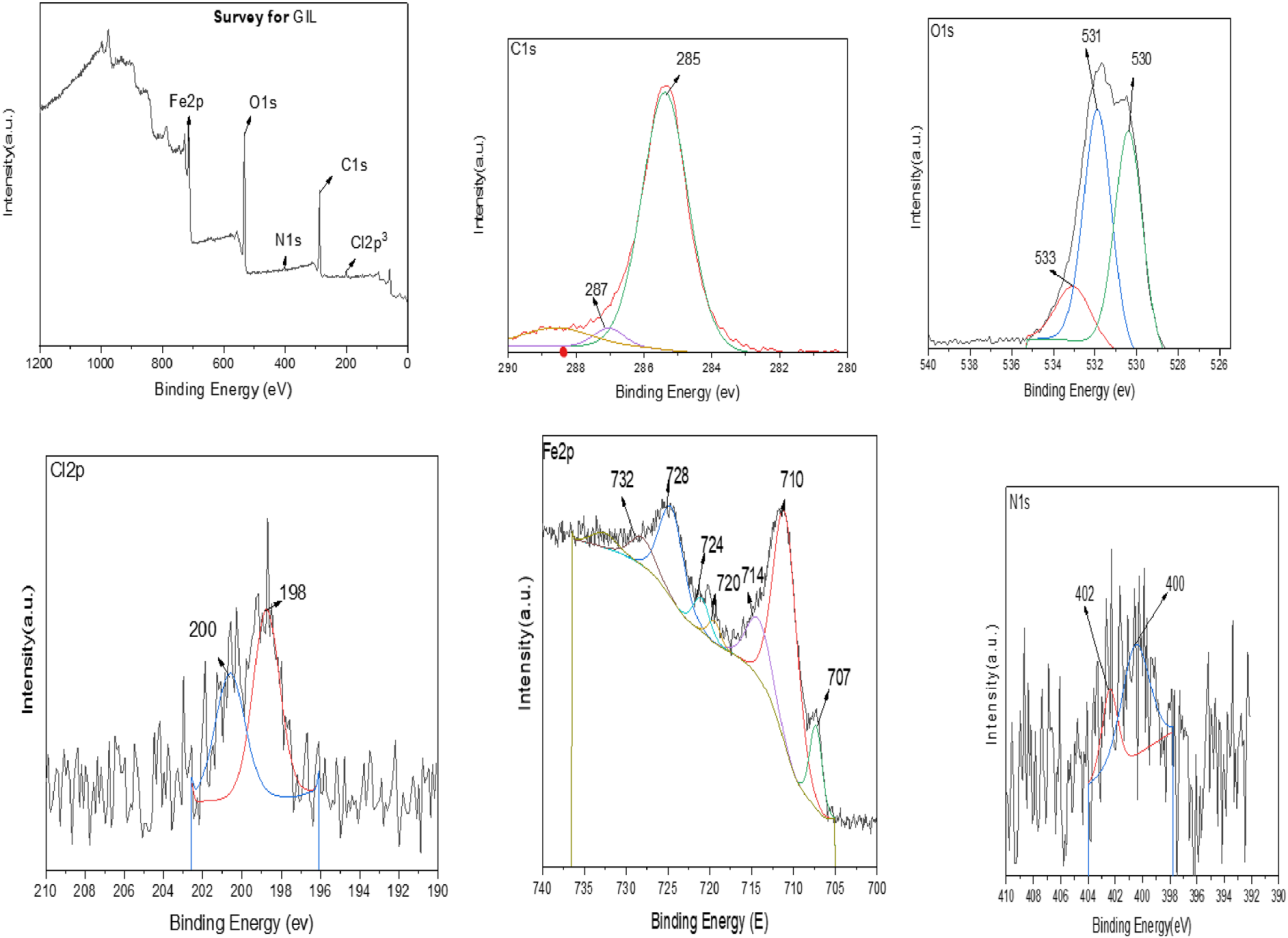
XPS spectra of the CS immersion in 1.0 M HCl in the Present of GIL inhibitor.

Corroded samples surface in the absence and presence of the corrosion inhibitor were also examined using the XPS technique which serves to analyze and predict the chemical composition of the outermost layer of the surface. The peak in the wide scan spectrum corresponds well with Fe element of the alloy. Figures 9, 10 and Table 6 show the XPS spectra of the synthesized GIL. XPS spectra in the absence of the corrosion inhibitor (Fig. 9) reveal peaks for O1s, Cl2p^3^ and Fe2p which indicates the formation of corrosion products. In the presence of the corrosion inhibitor, XPS spectra reveal the presence of N1s peak denoting the presence of the corrosion inhibitor on the surface of the alloy as shown in Fig. 10. From the full XPS survey of (Fig. 10), it can be concluded that the synthesized sample contained Fe (712 eV), O (532 eV), C (286 eV), Cl (200 Ev) and N (401eV) further confirming the successful formation of the GIL.

**Table 6:** Surface composition (Atom%) of CS after 24 h of immersion in 1 M HCl in the absence and presence of GIL.

Element	Blank	Blank + GIL
	Atom%	Atom%
O1s	38.79	34.38
Fe2p	12.34	10.23
C1s	42.34	51.03
Cl2p^3^	5.77	3.16
N1s	0.76	1.19
Total	100	100

To further understand the bonding formation, the high-resolution spectra of Fe, C, and N atoms were recorded. The high-resolution Fe2P spectrum can be deconvoluted into two peaks centered at 724 and 720 eV, suggesting the existence of GIL moieties. The high-resolution C1s spectrum and its deconvolution result is also displayed, and we can see that there were three different chemical environments for carbon atoms. The deconvoluted peaks of 285 and 287 eV corresponded to C–N bonding, respectively. The figure also shows the high-resolution N1s spectrum and the complexed at 400 eV corresponded to the nitrogen atom on the rings of the formed GIL.

### AFM analysis

**Figure 11:**
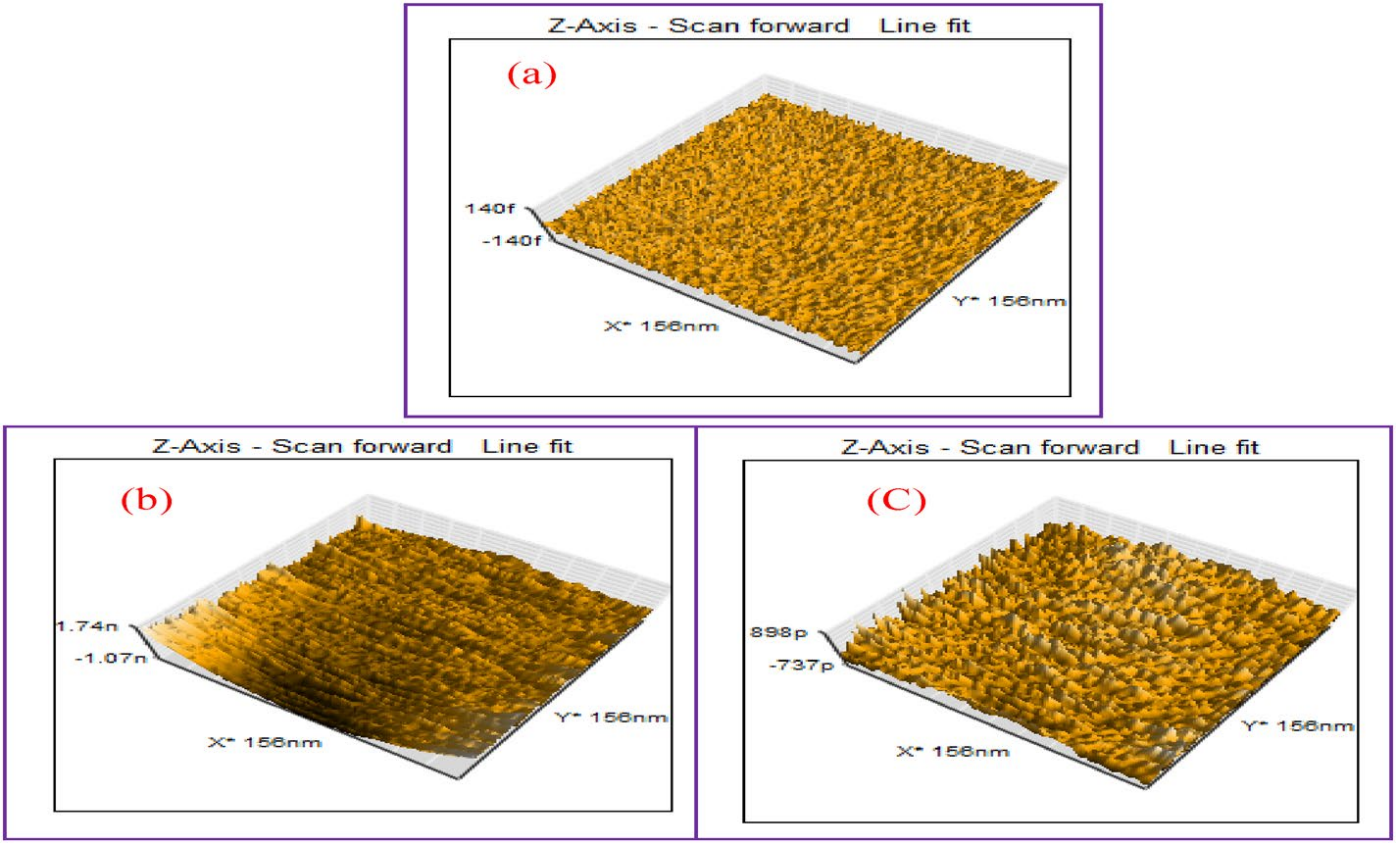
AFM 3D spectroscopy of various carbon steel specimen, (**a**) polished sample, (**b**) after immersion in 1.0 M HCl and (**c**) after immersion in blank with 200 ppm GIL compound at 298 K.

The 3D AFM pictures presented in Fig. 11a–c illustrate the surface features of abraded specimens. Specifically, the specimens examined include plain carbon steel before immersion, as well as after exposure to 1.0 M HCl without and with GIL-H (200 ppm) for a duration of 24 h at a temperature of 298 K. Figure 11b presents a notably uneven surface marked by substantial valleys stemming from carbon steel loss. Nevertheless, the highest points indicate the existence of diverse corrosion products^54^. The roughness average (Sa) values for abraded plain carbon steel were measured at 23.839 FM, rising to 259.1 pm without the inhibitor, and decreasing to 192.46 pm when the inhibitor was present. The roughness parameters of the surface were meticulously measured using AFM and are extensively documented in Table 7. In summation, the images and measurements unequivocally support the effectiveness of GIL-H in providing carbon steel surfaces with anti-corrosive properties under exposure to a 1.0 M HCl acid environment.

**Table 7:** AFM data for Carbon steel polished sample, after immersion in 1.0 M HCl and after immersion in blank with 200 ppm GIL compound at 298 K.

Parameters	Polished	Blank	GIL-H
The roughness averages. (Sa)	23.839 fm	259.1 Pm	192.46 pm
The mean value (Sm)	− 19.414 fm	− 19.317 fm	− 19.275 fm
The root mean square (Sq)	28.982 fm	364.17 pm	247.4 pm
The valley depth (Sv)	− 110.85 fm	− 1188.2 pm	− 1573.4 pm
The peak height (Sp)	91.572 fm	1939.4 pm	1.296 nm
The peak-valley height (Sy)	202.42 fm	3127.6 pm	2869.5 pm

## Theoretical study

**Figure 12:**
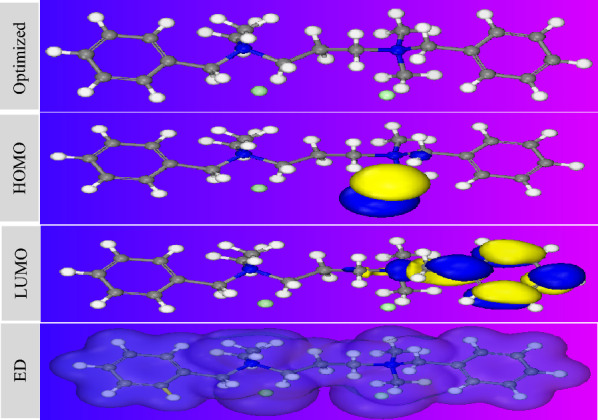
Optimized structures, distribution of orbitals (HOMO and LUMO) and ED of the investigated inhibitor.

To enhancement the chemical and electrochemical explanation for CS corrosion inhibition mechanism by the inhibitor, DFT was performed. We can expect the structure reactivity of the inhibitor using DFT method with DMOL_3_ module. The computational study involved in this work was done with BIOVIA Materials Studio 17.1.0.48 software from Accelrys, Inc. The frontier molecular orbitals (i.e., E_HOMO_ and E_LUMO_) can be correlated with various quantum chemical parameters such as energy gap in eV (ΔE = E_LUMO_ − E_HOMO_), global hardness (η), and electronegativity (χ) as in Table 8. After optimization process, electron densities are imported over the reactive sites of the inhibitor to give the HOMO, LUMO and electron density that shown in Fig. 12. The HOMO and the LUMO are located over nucleophilic and electrophilic centers, respectively which considered more active centers in the prepared inhibitor having high electron density^26^. In another meaning, the donation and accepting abilities of the inhibitor depend on the energy values of HOMO and LUMO, respectively. So, the electron donation process from the inhibitor to partially field 3d orbital of iron surface was done by the presence of Cl^−^ ions which considered as a nucleophilic center in the prepared inhibitor, HOMO. Moreover, the electron accepting from the filled 3d orbitals of iron surface, π-back donation, to the under studied inhibitor could be represent by LUMO which distributed over benzyl group and tertiary amine that represent the electrophilic center. The energy barrier layer of inhibitor molecules over the CS surface improves by the existence of electron density regions. On the other hand, the stability of Fe inhibitor complex and the chemical reactivity of any organic inhibitor could be obtained from ΔE values. Lower values of ΔE_gap_ give a higher tendency to remove electron from HOMO, enhancing adsorption probability on the metallic surface^55^. The E_gap_ values calculated from the energy difference between E_HOMO_ and E_LUMO_ are shown in Table 8 and Fig. 12. From Table 8, we can see that the adsorption priority of the prepared. It is well known that ΔE_gap_ values were associated with chemical hardness and softness parameters which related to chemical reactivity and stability of the molecules. The obtained data showed the inhibitor with higher chemical reactivity; therefore, it is more feasible to bind effectively on CS surface^55^. Fraction of electron transfer (∆N) parameter displays the efficiency of electron donor–acceptor interaction phenomena. ∆N is a function of the calculated electronegativity of GNs according to the following Eq. (9):

9 $$ \Delta {\text{N}} = \left( {\left( {\chi_{{{\text{Fe}}}} - \chi_{{({\text{inh}}.)}} } \right)} \right)/{2}\left( {\eta_{{{\text{Fe}}}} + \, \eta_{{({\text{inh}}.)}} } \right), $$

where χ_Fe_ is electronegativity of CS^56^. η _(inh.)_ and η_Fe_ symbol chemical hardness value of inhibitor and metal, respectively. As it is reported previously, the value of ∆N indicated the electron sharing from the inhibitor active centers to partially filled 3d orbital. From the results, it can be concluded that, the inhibitor has many active centers such as N atoms which enhance the inhibitor adsorption at CS/solution interface^56^.

**Table 8:** Quantum chemical parameters of the investigated inhibitor.

	*E*_HOMO_ (eV)	*E*_LUMO_ (eV)	Δ*E* (eV)	Ƞ (eV)	*E*_(b→d)_ (eV)	*X* (eV)	Δ*N*
*GIL*	− 0.1293	− 0.0532	0.0761	0.038	− 0.00951	0.0912	62.138

## Inhibition mechanism

**Figure 13:**
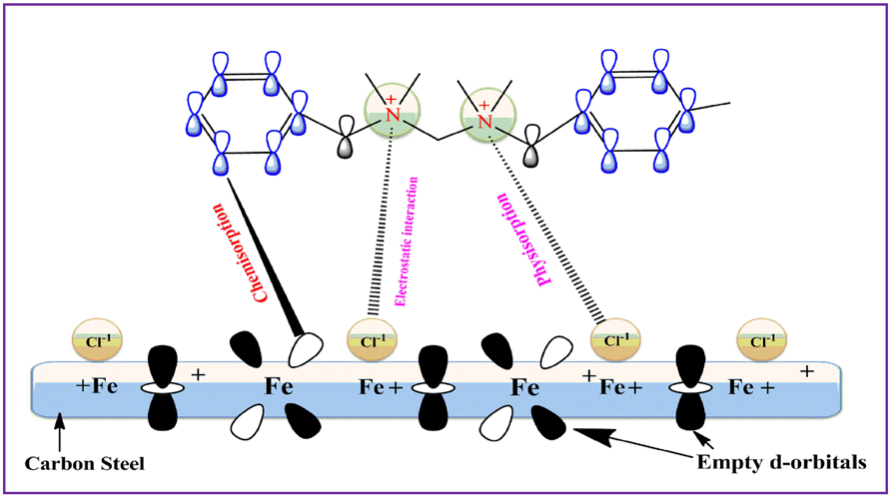
A diagrammatically scheme presenting the adsorption mechanism of the GIL active sites onto carbon steel surface.

According to the findings, carbon steel's corrosion rate can be slowed by raising the concentration of inhibitors in the acidic environment. Adsorption of corrosion inhibitor molecules onto carbon steel's surface halts the corroding process. Both chemical and physical processes can lead to adsorption. To enhance chemical adsorption, coordination-type bonds are formed between the electron pairs of heteroatoms, such as N or π electrons, and the empty d-orbitals of Fe^57^. Electrostatic repulsion between a negatively charged surface and a positively charged inhibitor results in physical adsorption. Figure 13 illustrates the significance of counter anions Cl^-^ in the adsorption process of the Gemini ionic liquid. Adsorption of counter anions, which turns the positively charged carbon steel surface into a negatively charged one Cl^-^, is responsible for the observed effects. The two positively charged quaternary nitrogen atoms (N^+^) can interact electrostatically with the negatively charged carbon steel surface^58^. The presence of anions between the protective film layer and the positively charged inhibitor segment is evidence that the present interaction can produce such a layer. The development of a coordination-type bond has been hypothesized to be responsible for the end of the dissolution processes on the surface of carbon steel that occur under anodic circumstances^59^. This bond arises between the vacant d-orbital of iron Fe and the unoccupied electron pairs originating from the nitrogen heteroatoms, along with the π electrons. Table 9 compares some of the inhibitors we investigated with those from the literature that are comparable in terms of chemistry and active function groups.

**Table 9:** Comparison of the Inhibition Efficiency of our investigated inhibitors with other inhibitors from the literature.

Inhibitor #	Electrolyte	Alloy or metal	Max achieved η_pp_ %	RF
N-(4-((4-(pyridin-2-yl)piperazin-1-yl)methyl) phenyl) quinoline-6-carboxamide	1.0 M HCl	Mild steel	65.17	^60^
*1-((4-ethylpiperazin-1-yl)(phenyl)methyl) thiourea*	0.5 M HCl	Mild steel	76.81	^61^
*7-((4-methylpiperazin-1-yl)methyl) quinolin-8-ol*	1.0 M HCl	C35E steel	86.3	^62^
*N1, N3 – dibenzyl—N1, N1, N3, N3—tetramethylpropane-1,3-diaminium tetrafluoroborate*	1.0M HCl	Brass alloy	81.40	^25^
*N1, N1, N3, N3-tetramethyl-N1, N3-bis (4-methyl benzyl) propane-1,3-diaminium tetrafluoroborate*	1.0 M HCl	Brass alloy	92.54	^25^
GIL-H	1.0 M HCl	Carbon Steel	87.60	This work

## Conclusion

The following sentences underscore the significant outcomes of the ongoing study:

1. This research aims to assess the effectiveness of the Gemini ionic liquid (GIL-H) as a corrosion inhibitor for carbon steel in the presence of a 1.0 M HCl solution, utilizing both chemical and electrochemical analyses. The Gemini ionic liquid synthesized demonstrated a remarkable overall inhibition efficiency of 94.8%.
2. Analysis of polarization data indicated that Gemini ionic liquids (GIL-H) functioned as a mixed inhibitor, primarily utilizing cathodic inhibition mechanisms.
3. The adsorption isotherm between the ionic liquid inhibitor and carbon steel conforms to the Langmuir model, suggesting monolayer formation on the material's surface. The calculated Δ G_ads_ value of -38.82 robustly supports physio-chemisorption predominance in this process.
4. The research effectively confirmed the existence of inhibitor molecules adhering to the surface of carbon steel, employing sophisticated methods such as (AFM), (SEM), and (EDX). The formation of molecular adhesion leads to the development of a resilient protective barrier, effectively shielding the carbon steel from the deleterious impacts of corrosive chemicals.

## Data Availability

The data that support the findings of this study are not publicly available because it is a part of a comprehensive study but available from the corresponding author on reasonable request.
